# Delayed Presentation of Hemichorea in Diabetic Striatopathy

**DOI:** 10.7759/cureus.30219

**Published:** 2022-10-12

**Authors:** Kai Xiong Lim, That Khaing Zin, Zijun Yu, Wee Ming Peh

**Affiliations:** 1 Internal Medicine, Sengkang General Hospital, Singapore, SGP

**Keywords:** hyperkinetic movement, chorea, diabetes mellitus, diabetic ketoacidosis, diabetic striatopathy

## Abstract

Diabetic striatopathy is a rare condition associated with poorly controlled diabetes that can present as hyperkinetic movements. A 70-year-old Asian female was newly diagnosed with type 2 diabetes mellitus complicated by diabetic ketoacidosis when she presented with lethargy and confusion. Computed tomography and magnetic resonance imaging of the brain performed for the patient showed incidental isolated radiological features of diabetic striatopathy, even though she did not have any hyperkinetic movements. After intensive glycemic control, the patient paradoxically developed a delayed presentation of hemichorea two weeks later. Pathological findings in diabetic striatopathy suggest the contributing role of vascular microangiopathy, similar to the changes seen in proliferative diabetic retinopathy. In order to avoid precipitating hyperkinetic movements, a less intensive diabetic control could be considered for asymptomatic patients with isolated radiological features of diabetic striatopathy. This is especially important in patients at higher risk of the condition.

## Introduction

The association between hyperkinetic movement and elevated blood glucose levels was first described in 1960 by Bedwell [1]. Five decades later, the term “Diabetic Striatopathy” (DS) was introduced to define a syndrome of movement disorders in diabetic patients with corresponding striatal hyperintensity on T1-weighted (T1w) magnetic resonance imaging (MRI) [2].

We discuss a case of an elderly Asian female with newly diagnosed type 2 diabetes mellitus complicated by diabetic ketoacidosis. She initially had incidental radiological features of diabetic striatopathy without hyperkinetic movements. However, she subsequently developed hemichorea after aggressive treatment of hyperglycemia.

We review the postulated pathophysiology of diabetic striatopathy and explore the possible reasons for the paradoxical delayed hyperkinetic movement when glycemic levels were well-controlled. This could guide physicians’ approach in the treatment of hyperglycemia in patients with isolated radiological evidence of diabetic striatopathy, as well as patients who are at higher risk of DS. This case report also aims to raise awareness of diabetic striatopathy among physicians regarding this poorly understood rare condition with variable radiological and clinical manifestations [3].

## Case presentation

A 70-year-old Asian female presented with lethargy and confusion and was newly diagnosed with type 2 diabetes mellitus (DM) complicated by diabetic ketoacidosis (DKA). Initial investigations showed serum glycated hemoglobin of 16.3%, serum glucose of 46.1 mmol/L, effective serum osmolality of 312 mOsm/kg, pH of 7.30, serum bicarbonate of 11.4 mmol/L, and serum ketones level above the laboratory detection limit of 6.0 mmol/L. She had a past medical history of hypertension, hyperlipidemia, and gout. There was no family history of movement disorder.

**Figure 1:**
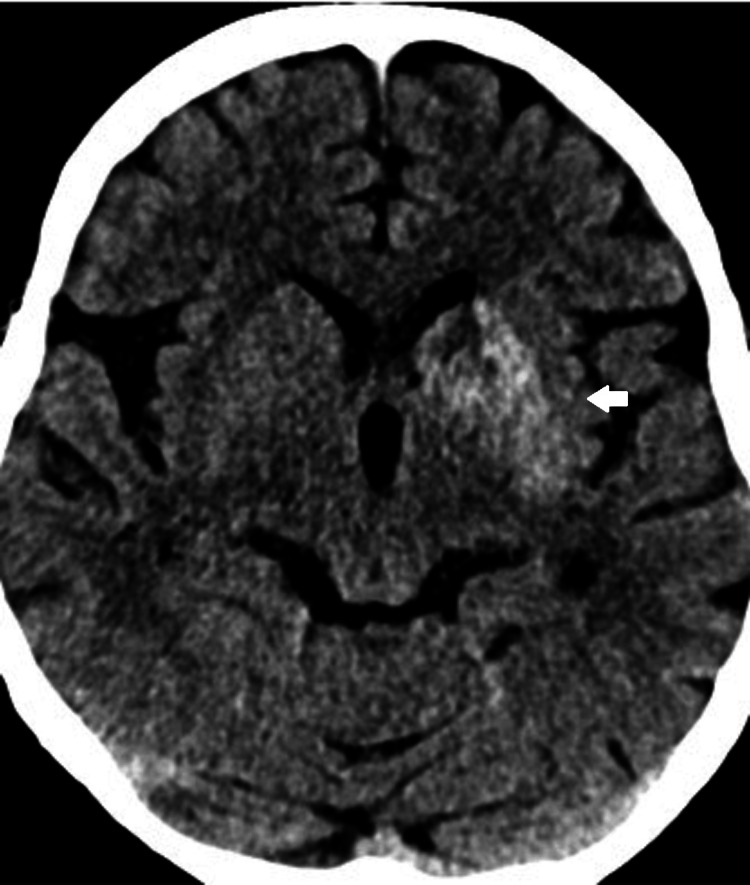
Non-contrasted CT brain showed hyperdensity at the left basal ganglia

**Figure 2:**
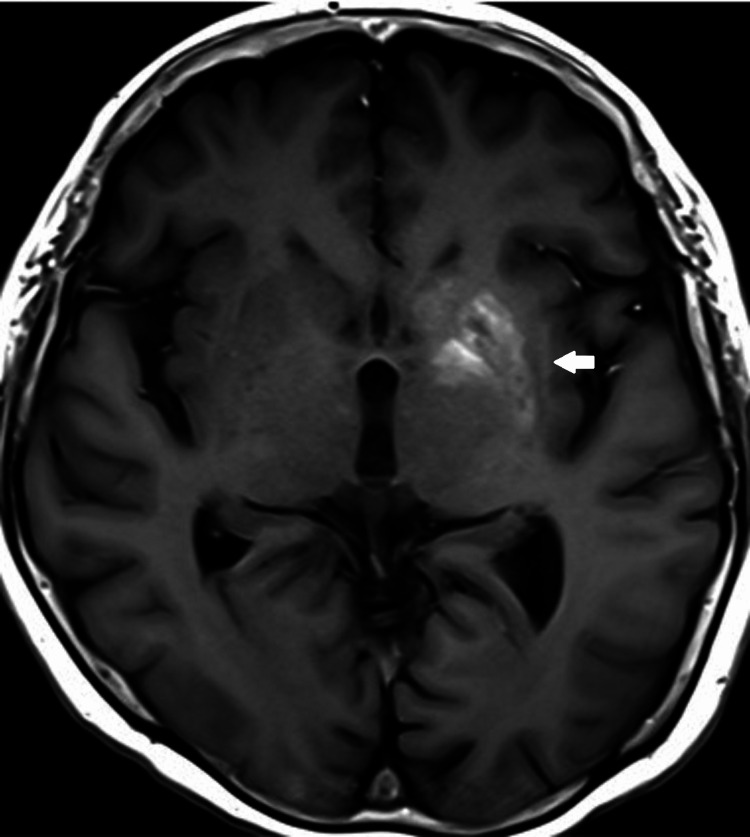
MRI Brain - Intrinsic T1W hyperintensity of the left basal ganglia

**Figure 3:**
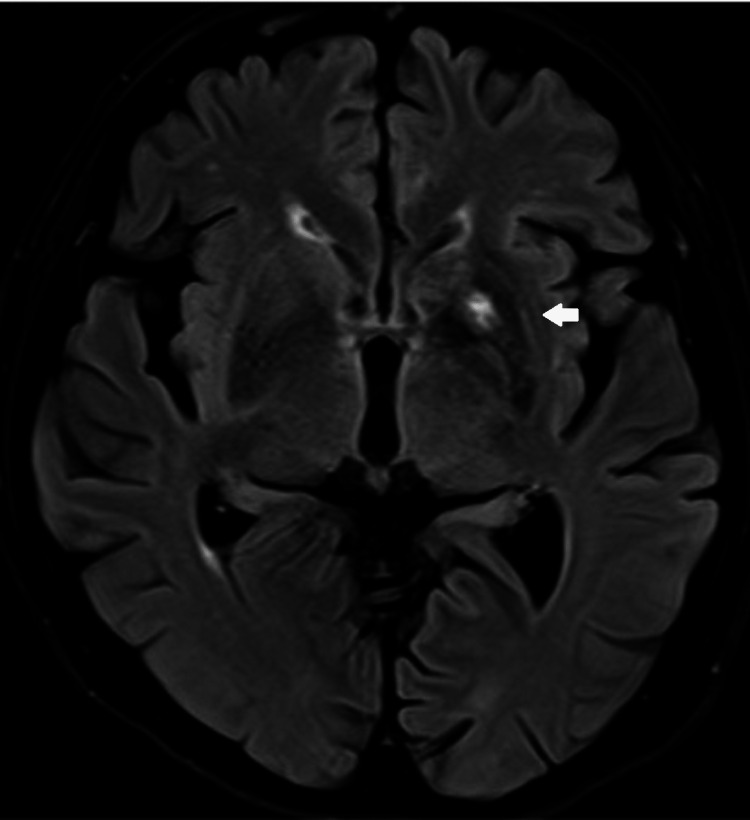
MRI Brain - Focal fluid attenuated inversion recovery (FLAIR) hyperintensity at the left lentiform nucleus

A non-contrasted brain computed tomography (CT) done for the evaluation of altered mental status showed left basal ganglia hyperdensity (Figure 1). A subsequent MRI brain showed intrinsic T1w hyperintensity in the left basal ganglia involving the left caudate head and lentiform nucleus (Figure 2) with focal fluid attenuated inversion recovery (FLAIR) hyperintensity at the left lentiform nucleus (Figure 3), consistent with DS. The patient's Glasgow Coma Scale was 15, and her neurological examination was normal for tone, reflexes, power, and sensation. She did not have any involuntary movements.

The DKA was treated with intravenous Actrapid and fluid resuscitation, with the resolution of the DKA achieved within 10 hours. Following the DKA resolution, the insulin regime was converted to subcutaneous insulin glargine for basal coverage and subcutaneous insulin glulisine for prandial coverage. The patient’s diabetic regime was further adjusted during the admission based on capillary blood glucose readings. Her diabetic medication upon discharge from the hospital was insulin glargine 18 units once daily, metformin 500mg twice daily and sitagliptin 100 mg every morning. There were no hypoglycemia episodes during this admission.

**Figure 4:**
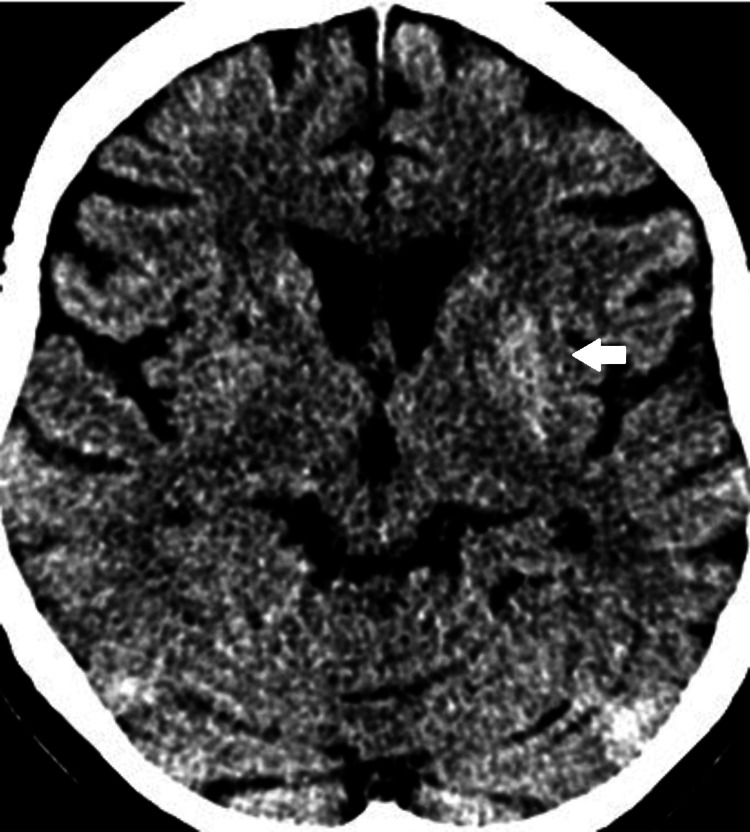
Non-contrasted CT brain showed residual faint hyperdensity of the left basal ganglia

**Figure 5:**
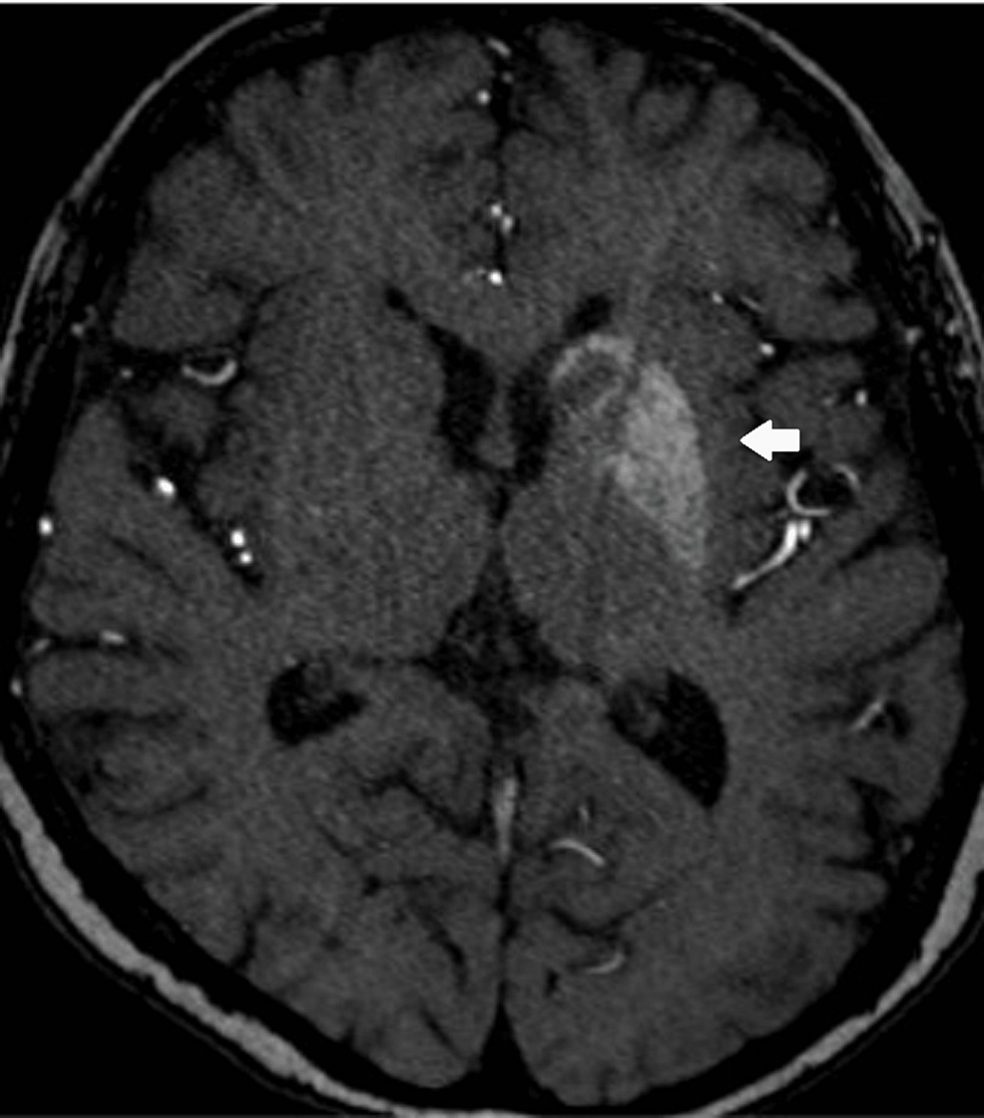
MRI stroke protocol - Time-of-flight magnetic resonance angiography showed persistent T1w hyperintensity of the left basal ganglia

**Figure 6:**
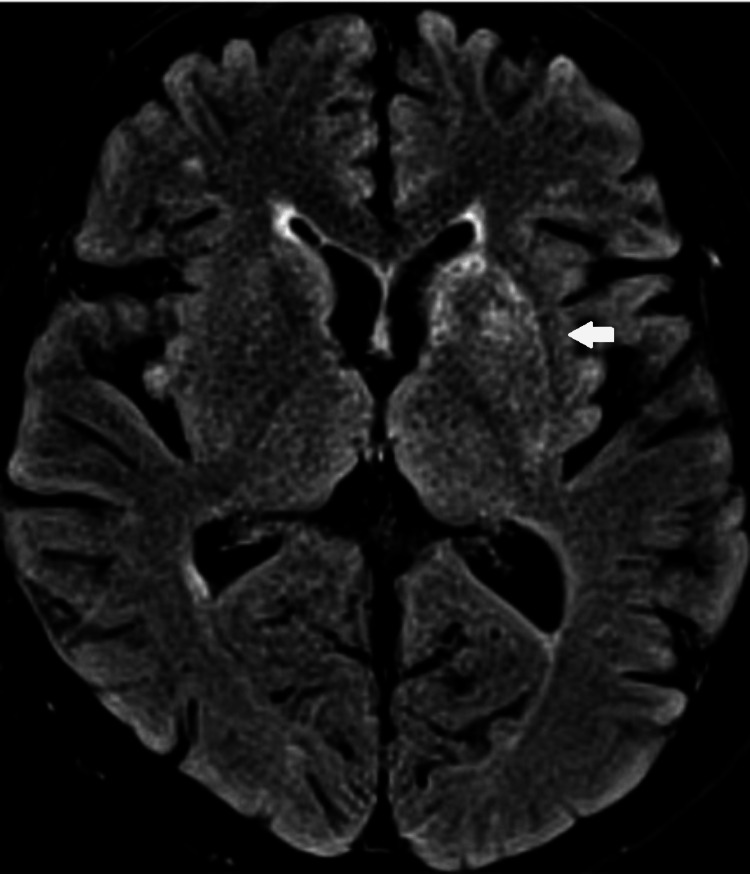
MRI stroke protocol - Reduced focal fluid attenuated inversion recovery (FLAIR) hyperintensity at the left lentiform nucleus

The patient presented two weeks later with right-sided hemichorea. Her mentation was normal, and she had normal tone, reflexes, power, and sensory examination. Non-contrasted CT brain showed residual faint hypodensity over the left basal ganglia (Figure 4). MRI brain stroke protocol showed persistent T1w hyperintensity of the left basal ganglia (Figure 5), with reduced focal FLAIR hyperintensity at the lentiform nucleus (Figure 6). There was no acute infarct.

Differentials for hemichorea were excluded. Serum caeruloplasmin, liver function test, thyroid function test, antinuclear antibody, and anti-double stranded DNA antibody were normal.

During this hospitalization, there were episodes of asymptomatic hypoglycemia episodes. The lowest recorded capillary blood glucose was at 2.9 mmol/L. The patient did not report any episodes of hypoglycemia after the previous discharge from the hospital for DKA. In view of the overly tight glycemic control, the DM regime was adjusted to insulin glargine 12 units once daily and linagliptin 5 mg once daily. Serum glycated hemoglobin repeated three months after the initial assessment was 4.7%, suggesting that intensive control of DM was achieved rapidly.

The patient was started on tetrabenazine 12.5 mg twice daily for treatment of the hemichorea. Three months after the hemichorea onset, there was an improvement in symptoms with tetrabenazine although the hemichorea continued to persist. A follow-up MRI of the brain showed persistent T1w hyperintensity of the left basal ganglia with a further reduction of the focal FLAIR hyperintensity of the left lentiform nucleus. There were no further episodes of hypoglycemia.

## Discussion

Diabetic striatopathy is a recognized complication of uncontrolled diabetes mellitus that remains poorly understood. Hyperkinetic movement disorder with T1w hyperintensity in contralateral putamen without surrounding edema or mass effect, along with hyperglycemia is considered a pathognomonic feature. Cases demonstrate atypical laterality, have varying temporal relations to hyperglycemia, and even clinical-radiological dissociation. This has prompted a call for further classification into symptomatic DS, clinically isolated DS, or radiologically isolated DS [4]. This could enable researchers to better understand the pathological mechanisms.

A systematic review of 176 cases [3] suggests that DS predominantly occurs in patients with type 2 DM with non-ketotic hyperglycemia and unilateral hyperkinetic movement disorders affecting limbs. The average age of presentation for DS was 67.6. DS is more common in females, with a male-to-female ratio of 1:1.7 [3]. DS is rare in patients with type 1 diabetes or in patients with ketotic hyperglycemia. It rarely involves the face or trunk and is usually unilateral rather than bilateral. In most cases, the hyperkinetic movement started during hyperglycemia, with average serum glucose of 22.97mmol/L and serum glycated hemoglobin of 13.1% [3]. In rare cases, the onset of DS in patients is when glycemic levels were controlled [2,5-7]. Neuroimaging abnormalities may precede hyperkinetic movement [8], like in our patient’s case, or may be absent. A mismatch in the correlating laterality of the lesion on neuroimaging and the affected side of abnormal movements have also been observed [4]. This suggests that radiologic lesions alone are inadequate to cause hyperkinetic movement, and triggers may be related to glycemic fluctuations.

Interestingly, our patient had radiological features of basal ganglia changes without hyperkinetic movement at the initial presentation. However, her hemichorea commenced after the rapid correction of her hyperglycemia. This mimics the clinical course in diabetic retinopathy, where tight glycemic control can result in paradoxical deterioration. Indeed, the retina and striatopallidal regions are similar in their vulnerability to vascular damage during energy crises, owing to their scarce collateral circulation [9]. Pathological findings in DS suggest the contributing role of vascular microangiopathy, reminiscent of changes seen in proliferative retinopathy [2]. The severity of retinopathy might be associated with an increased risk of hyperkinetic disorders in DS [10].

The similarities between diabetic retinopathy and DS may guide clinicians to adopt a less intensive glycaemic control regime to reduce the risk of precipitating hyperkinetic movement in those with radiologically isolated DS who are at higher risk. Another proposed mechanism of DS is the rapid depletion of cerebral gamma-aminobutyric acid (GABA) levels in non-ketotic hyperglycemia [11]. Our case adds to existing case reports of ketotic hyperglycemia in DS and serves to argue against this mechanism.

The prognosis is favorable, and most patients achieve partial to complete resolution of symptoms either with glucose control or anti-chorea medications. The median recovery time was two days in those with only glucose control and fourteen days in those who needed anti-chorea medications [3]. The presence of hyperdense blood products on the initial CT brain may predict a more prolonged course [9]. The patient continued to have right-sided hemichorea and this was associated with a persistent T1w hyperintensity of the left basal ganglia. We postulate that persistent radiological changes may also predict the persistence of abnormal movements.

## Conclusions

Diabetic striatopathy is a rare complication of hyperglycemia that remains poorly understood. Intensive glycemic control may cause paradoxical worsening or precipitate symptoms in DS, similar to diabetic retinopathy. This may shed light on the underlying pathophysiology in which vascular microangiopathy of the striatopallidal region is likely to play a key role. Hence, less intensive diabetic control could be considered for patients with asymptomatic isolated radiological features of DS, and those who are at higher risk of DS. Risk factors to be aware of include patients who are elderly, female, and have type 2 diabetes with high glycated hemoglobin levels. Given that DS could present in many different ways, it may be worthwhile to classify DS by its varying radiological and clinical presentations to facilitate future research. With further classification and a better understanding of the disease, we hope to raise awareness of this condition among physicians.

## References

[1] BE SF (1960). Some observations on hemiballismus. Neurology.

[2] Abe Y, Yamamoto T, Soeda T (2009). Diabetic striatal disease: clinical presentation, neuroimaging, and pathology. Intern Med.

[3] Chua CB, Sun CK, Hsu CW, Tai YC, Liang CY, Tsai IT (2020). "Diabetic striatopathy": clinical presentations, controversy, pathogenesis, treatments, and outcomes. Sci Rep.

[4] Dubey S, Biswas P, Ghosh R, Chatterjee S, Kanti Ray B, Benito-León J (2022). Neuroimaging of diabetic striatopathy: more questions than answers. Eur Neurol.

[5] Lin CJ, Huang P (2017). Delayed onset diabetic striatopathy: hemichorea-hemiballism one month after a hyperglycemic episode. Am J Emerg Med.

[6] Taboada GF, Lima GA, Castro JE, Liberato B (2013). Dyskinesia associated with hyperglycemia and basal ganglia hyperintensity: report of a rare diabetic complication. Metab Brain Dis.

[7] Shivkumar V, Nemade D (2022). Hemichorea-hemiballism as a delayed manifestation of hyperglycemia: a case report. The Neurohospitalist.

[8] Higa M, Kaneko Y, Inokuchi T (2004). Two cases of hyperglycemic chorea in diabetic patients. Diabet Med.

[9] Lizarraga KJ, Adams D, Post MJ, Skyler J, Singer C (2017). Neurovascular uncoupling after rapid glycemic control as a trigger of the diabetic-uremic striatopallidal syndrome. Parkinsonism Relat Disord.

[10] Lizarraga KJ, Chunga N, Yannuzzi NA, Flynn HW Jr, Singer C, Lang AE (2020). The retina as a window to the basal ganglia: systematic review of the potential link between retinopathy and hyperkinetic disorders in diabetes. Parkinsonism Relat Disord.

[11] Das L, Pal R, Dutta P, Bhansali A (2017). "Diabetic striatopathy" and ketoacidosis: report of two cases and review of literature. Diabetes Res Clin Pract.

